# Fuzzy Rule-Based Classification System for Assessing Coronary Artery Disease

**DOI:** 10.1155/2015/564867

**Published:** 2015-09-13

**Authors:** Reza Ali Mohammadpour, Seyed Mohammad Abedi, Somayeh Bagheri, Ali Ghaemian

**Affiliations:** ^1^Department of Biostatistics, Faculty of Health, Diabetes Research Center, Mazandaran University of Medical Sciences, Sari 4817844718, Iran; ^2^Department of Radiology, Faculty of Medicine, Mazandaran University of Medical Sciences, Sari 4817844718, Iran; ^3^Department of Biostatistics, Faculty of Health, Zabol University of Medical Sciences, Zabol, Iran; ^4^Mazandaran Heart Center, Mazandaran University of Medical Sciences, Sari 4817844718, Iran

## Abstract

The aim of this study was to determine the accuracy of fuzzy rule-based classification that could noninvasively predict CAD based on myocardial perfusion scan test and clinical-epidemiological variables. This was a cross-sectional study in which the characteristics, the results of myocardial perfusion scan (MPS), and coronary artery angiography of 115 patients, 62 (53.9%) males, in Mazandaran Heart Center in the north of Iran have been collected. We used membership functions for medical variables by reviewing the related literature. To improve the classification performance, we used Ishibuchi et al. and Nozaki et al. methods by adjusting the grade of certainty *CF*_*j*_ of each rule. This system includes 144 rules and the antecedent part of all rules has more than one part. The coronary artery disease data used in this paper contained 115 samples. The data was classified into four classes, namely, classes 1 (normal), 2 (stenosis in one single vessel), 3 (stenosis in two vessels), and 4 (stenosis in three vessels) which had 39, 35, 17, and 24 subjects, respectively. The accuracy in the fuzzy classification based on if-then rule was 92.8 percent if classification result was considered based on rule selection by expert, while it was 91.9 when classification result was obtained according to the equation. To increase the classification rate, we deleted the extra rules to reduce the fuzzy rules after introducing the membership functions.

## 1. Introduction

In the past years, fuzzy if-then rule-based systems were used basically to control problems, while nowadays they are mainly applied in classification tasks [1–7]. There are many methods for automatically generating and learning the fuzzy if-then rules from numerical data for pattern classification problems [2, 4, 5, 8, 9].

The concepts of linguistic statement have been introduced by Zadeh [10] and it is crucial that we see each attribute as linguistic value showed by fuzzy numbers with trapezoidal membership function [11, 12].

After generating the volunteer rule, a set of rules must be chosen to structure the rule based on the classifier. In this paper, we assume a number of prespecified fuzzy sets given by expert's knowledge for each input attribute [13]. The performance of resulting classifiers can be enhanced by using rule weighting [14]. Therefore, our study can have effect on generating, weighting, and selecting rules based on expert opinions or systems outputs.

Therefore, the total number of fuzzy if-then rules generated by partitioning each attribute into *K* fuzzy subsets in an *n*-dimensional pattern classification is defined as *K* ^*n*^ [4, 5, 15]. In this work, we adjusted the grade of certainty of fuzzy if-then rules when there was misclassification of an input pattern in an error-correction method [15].

Coronary artery disease (CAD) is the most common cause of mortality in worldwide population and has a high prevalence of mortality rate in both developing and developed countries [16]. Many risk factors such as age, sex, high blood pressure, diabetes, obesity, smoking, family history of coronary artery disease, and cholesterol (LDL) have essential role in CAD [17]. Some risk factors such as sex and diabetes are crisp, whereas others are fuzzy sets.

The gold standard method for the diagnosis of CAD is coronary angiography (CA). Since CA is a costly and invasive procedure needing technology and high level of technical experience, it cannot be used to screen large population [18–20]. Therefore, noninvasive alternative methods for coronary angiography are necessary. A number of noninvasive CAD diagnosis methods have been proposed in literature [21], namely, exercise stress test and Single Photon Emission Computed Tomography (SPECT) or scintigraphy and Echo. However, the diagnosis accuracy of these tests is not as high as that of coronary angiography. Some studies showed that these medical tests do not have an accurate result and fuzzy set theory can enhance their accuracy [22–25]. Moreover, the diagnosis accuracy of a combination of the results of noninvasive clinical tests and other clinical-epidemiological attributes has been improved by using fuzzy models [26]. Fuzzy systems have been proposed and used to determine the cardiovascular diseases and to assess the risk factors due to the ambiguity and uncertainty of the diagnostic process [27, 28].

Fuzzy rule-based classification system is one of the fuzzy systems. In recent years, fuzzy classifier methods have been commonly used in medical diagnosis. In the literature there are a lot of studies that have worked on the classification of medical data (on cancer and cardiology) using fuzzy classifiers [29, 30]. In 2004, Vig et al. employed fuzzy set theory for the diagnosis of CAD [31]. Allahverdi et al. designed a fuzzy expert system to determine coronary heart disease risk [32] and P. Srivastava and A. Srivastava conducted similar study in India [33] and some of investigators studied improving fuzzy decision systems for CAD diagnosis.

Clinical importance or relevance of features (input variables) in cardiology diagnostic tests can introduce weights for interpretable fuzzy rule selection. CAD diagnosis is a complex and important problem. Some of rules are clinically acceptable and desirable by physicians. In this paper, we added the result of MPS to other attributes for generating fuzzy rules according to physician knowledge and supervised classification with labeled data. Data set is classified using Ishibuchi et al. [34–36] weighted fuzzy rule-based classifier to diagnose CAD and 3 levels of severity of CAD.

The aim of this study was to determine the accuracy of fuzzy rule-based classification that could noninvasively predict the CAD based on myocardial perfusion scan test and clinical-epidemiological variables.

After Introduction in Section 1 we continued, in Section 2, generating, learning, and weighting fuzzy if-then rules. Finally, in Sections 3 and 4, we showed application results and discussion.

## 2. Methods: Weighting Fuzzy Classification System

### 2.1. Fuzzy If-Then Rule

Let us suppose that our pattern classification problems are *m* training patterns *X*_*p*_ = (*X*_*p*1_,…, *X*_*pn*_), *p* = 1,…, *m* with *c*-class *n*-dimensional problem [5, 7, 30].

First, all attribute values of the given training pattern are transformed in the unit interval [0,1]. Thus, the pattern space into the *n*-dimensional pattern classification problem uses unit hypercube [0, 1]^*n*^ [5, 6, 34]. For an *n*-dimensional, *c*-class problem, we apply fuzzy if-then rule of the following form:

(1) Rule  Rj:  If  x1  is  Aj1  and  …  and   xn  is  Ajn  Then  class  Cj  with  CFj j=1,…,N,

where *R*_*j*_ is the label of the *i*th fuzzy if-then rule, *N* is the total number of fuzzy if-then rules, *X* = [*x*_1_,…, *x*_*n*_] is the pattern vector *n*-dimensional, *A*_*j*1_ presents antecedent fuzzy sets for the *i*th attribute, *C*_*j*_ represent a consequent class (i.e., one of the *c* classes), and *CF*_*j*_ is a certainty grade of the fuzzy if-then *R*_*j*_ [2, 5, 9, 13, 15, 34, 35].

As antecedent fuzzy sets we employ trapezoidal fuzzy sets, where we display other partitions of the unit interval into fuzzy sets [2, 36].

Using (1), we generate *N* fuzzy if-then rule that consists of two below steps. The first one is specifying membership function of antecedent fuzzy sets and the second one is determining consequent class *C*_*j*_ and certainty grade *CF*_*j*_ of the fuzzy rule *R*_*j*_ [2, 5, 15]. The antecedent part of the fuzzy if-then rules is initialized manually [2]. For each training pattern, the concept of a weight is applied. The weight of misclassified/rejected patterns is observed as a cost of misclassification or rejection. When a training pattern is misclassified, then the adjustment of fuzzy rules arises. In this study, we can determine both consequent class *C*_*j*_ and the grade of certainty *CF*_*j*_ for all rules of the following type.

**Step 1 .**

Calculate the compatibility grade of training patterns using product as T-norm:

(2) μjXp=μj1xp1×⋯×μjnxpn,p=1,2,…,m,

where *μ*_*ji*_(·) is the membership function of fuzzy sets *A*_*ji*_.

**Step 2 .**

For each class *h* can be calculated *β*_class *h*_(*R*_*j*_) according to

(3) $$\begin{array}{c} \beta_{\text{class}\;h}\left(\;R_{j}\;\right)=\underset{x_{p}\in \text{class}\;h}{\sum }\mu_{j}\left(\;x_{p}\;\right)\cdot w_{p}\;h=1,2,\ldots ,c. \end{array}$$

And *w*_*p*_ is the weight of the training pattern.

**Step 3 .**

Find class $\hat{h}$ that has largest sum of *β*_class *h*_(*R*_*j*_):

(4) $$\begin{array}{c} \beta_{\text{class}\;\hat{h}}\left(\;R_{j}\;\right)=\underset{1\leq l\leq c}{\max}\left\{\;\beta_{\text{class}\;l}\left(\;R_{j}\;\right)\;\right\}. \end{array}$$

Note that if two or more classes take the maximum value of (4), then the consequent class *C*_*j*_ of the fuzzy rule *R*_*j*_ cannot be individually determined, so *C*_*j*_ is also specified as *∅*. Let the grade of certainty *CF*_*j*_ of fuzzy rule *R*_*j*_ be *CF*_*j*_ = 0. If only class takes the maximum value, let *C*_*j*_ be class $\hat{h}$. The grade of certainty *CF*_*j*_ can be assigned as follows:

(5) $$\begin{array}{c} {CF}_{j}=\frac{\beta_{\text{class}\;\hat{h}}\left(R_{j}\right)-\overset{-}{\beta }}{\sum_{h}\beta_{\text{class}\;h}\left(R_{j}\right)}. \end{array}$$

With

(6) $$\begin{array}{c} \overset{-}{\beta }=\frac{\sum_{h\neq \hat{h}}\beta_{\text{class}\;h}\left(R_{j}\right)}{c-1} \end{array}$$

*c* is the total number of classes.

After generating *N* fuzzy if-then rules by (1), both the consequent class and the grade of certainty can be determined for all *N* rules; then a new pattern *X* = (*x*_1_,…, *x*_*n*_) according to the following procedure can be classified.

**Step 1 .**

For class *h*  (*h* = 1, …, *c*), calculate *α*_class *h*_(*X*) as  

(7) $$\begin{array}{c} \alpha_{\text{class}\;h}\left(\;X\;\right)=\max\left\{\;\mu_{j}\left(\;X\;\right)\cdot {CF}_{j}\;|\;C_{j}=h\;\right\}. \end{array}$$

**Step 2 .**

Find class *h* ^*∗*^ that has the largest sum of *α*_class *h*_(*X*):

(8) $$\begin{array}{c} \alpha_{\text{class}{\;h}^{*}}\left(\;X\;\right)=\underset{1\leq k\leq c}{\max}\left\{\;\alpha_{\text{class}\;k}\left(\;X\;\right)\;\right\}. \end{array}$$

When multiple classes take the same (maximum) value in (8), then the classification of *X* cannot be classified (i.e., *X* is left as an unclassifiable pattern); otherwise, a sign *X* to class *h* ^*∗*^ (i.e., *X*) is considered as a classifiable pattern by (8).

### 2.2. Learning Fuzzy If-Then Rules

In this section, we use a method for improving classification performance. It adjusts the grade of certainty *CF*_*j*_ of each rule in Nozaki et al. [37].

Distributed representation of fuzzy rules is a general concept which is not restricted within the field of pattern classification [38]. The total number of the distributed fuzzy rules can be reduced by removing some unnecessary rules from (1) since such selection of fuzzy rules may require a complicated procedure.

When a fuzzy rule is found, training patterns covered by these rules are removed or their weights are set to zero. Then another fuzzy rule is found and added to the rule set using the modified training set [39].

After generating rules by system, each rule was evaluated by expert for decreasing the rule space dimension. Number of rules inducted by systems was 545 rules which were then reduced to 144 by using expert rule selection. We assigned weights to rules in two cases separately.

When a training pattern was not successfully classified by the fuzzy if-then rule *R*_*j*_, its grade of certainty was decreased as

(9) $$\begin{array}{c} {CF}_{j}^{\text{new}}={CF}_{j}^{\text{old}}-\eta \cdot w_{p}\cdot {CF}_{j}^{\text{old}}. \end{array}$$

On the contrary, if the training pattern was correctly classified, we could reinforce the grade of certainty in the following manner:

(10) $$\begin{array}{c} {CF}_{*}^{\text{new}}={CF}_{*}^{\text{old}}+\eta \cdot w_{p}\cdot \left(\;1-{CF}_{*}^{\text{old}}\;\right), \end{array}$$

where *η* is a positive constant value. Let 0 ≤ *η* ≤ 1, and *w*_*p*_ is the weight of the pattern [36].

### 2.3. Cost Function

This section evaluates the performance of classification systems. Under the hypothesis that a weight is determined to each training pattern, we applied the weight of training patterns as a cost of misclassification. A cost function cost(*S*) of a fuzzy classification system *S* is defined as

(11) $$\begin{array}{c} \text{Cost}\left(\;S\;\right)=\overset{m}{\underset{p=1}{\sum }}w_{p}\cdot z_{p}\left(\;S\;\right), \end{array}$$

where *Z*_*p*_(*S*) is defined as duplex variable to the classification result of the pattern *x*_*p*_ by *S*: if *x*_*p*_ is correctly classified by *S*, then *Z*_*p*_(*S*) = 0, and if *x*_*p*_  is misclassified or rejected, *Z*_*p*_(*S*) = 1 and *m* is the total number of training patterns. This cost function as good as classification rate is used [36]. We employed four types of weight which were introduced by Nakashima et al. in 2005 [36].

In this paper, we randomly used training pattern *w*_*p*_ as *w*_*p*_ = 0.25, 0.5, 0.7, 0.9 and then we randomly assigned a weight to each training pattern. For example, we specified *w*_*p*_ as *w*_*p*_ = 0.25 if the training pattern *x*_*p*_ belonged to class 1 and *w*_*p*_ = 0.25, 0.5, 0.7, 0.9 if *x*_*p*_ belonged to classes 2, 3, and 4 and *w*_*p*_ as *w*_*p*_ = 0.5 if the training pattern *x*_*p*_ belonged to class 1 and *w*_*p*_ = 0.25, 0.5, 0.7, 0.9 if *x*_*p*_ belonged to classes 2, 3, and 4, and so forth.

## 3. Application Results

### 3.1. Patients

This study was a cross-sectional study in which characteristics, results of myocardial perfusion scan (MPS), and coronary artery angiography of 115 patients, 62 (53.9% male), in Mazandaran Heart Center (Iran) have been collected. The coronary artery disease data used in this paper contained 115 samples classified into four classes, namely, classes 1 (normal), 2 (stenosis in one single vessel), 3 (stenosis in two vessels), and 4 (stenosis in three vessels) having 39, 35, 17, and 24 subjects, respectively. The dataset included ten input variables (age, sex, diabetes, cholesterol level, triglyceride level, low density lipoprotein (LDL), systolic blood pressure, summed stress score (SSS), smoking, and genetic factor) and one output. We used membership functions for medical variables according to literature review. For these variables we took 3 fuzzy sets for age (young age, middle age, and old age), 3 fuzzy sets for the cholesterol level and triglyceride level (normal, borderline, and high), 3 fuzzy sets for LDL (normal, middle, and high), 4 fuzzy sets for systolic blood pressure (low, middle, high, and very high), and 4 fuzzy sets for SSS (normal, mild, moderate, and severe).

Sex had two stages (0 = male and 1 = female), smoking had two parts (0 = nonsmoking and 1 = smoking), and genetic factor diabetes had two sections (0 = no and 1 = yes).

**Figure 1:**
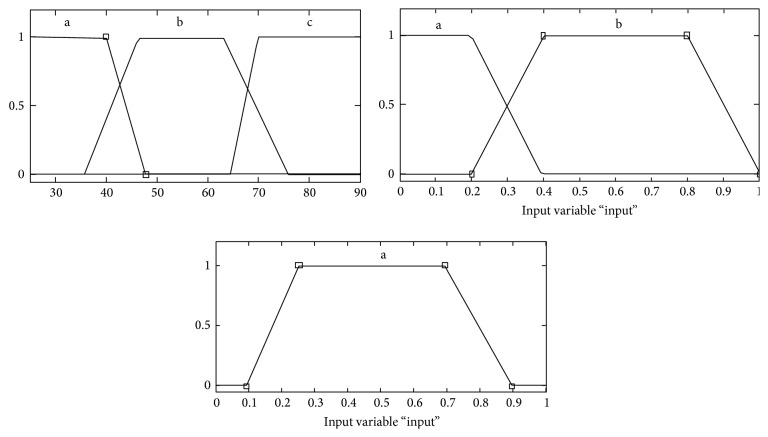
Membership function.

The type of membership functions for each fuzzy set applied was the trapezoidal membership function as shown in Figure 1 and membership functions of input variables were* defined by form in Table 1.*

### 3.2. Fuzzy Rule Base

The main part in fuzzy system is the rule base and the results in a fuzzy classification system depend on the fuzzy rules. This system includes 144 rules. Antecedent part of all rules has more than one part. In this study, we used linguistic variables including middle (M), high (H), very high (VH), low (L), young (Y), old (O), moderate (Mo), mild (Mi), severe (S), normal (NR), borderline (B), middle age (M), middle (mid), yes (Y), and no (N) for input variables.

The units of the used input variables were age, cholesterol (CL), triglyceride (TG), systolic blood pressure (SY), LDL, SSS, sex, diabetes (DI), genetic factor (GF), and smoking (SM).

Parts of the fuzzy rules are shown in Table 2. A series of 144 rules are formed.

After learning fuzzy if-then rules by training patterns, we used different weights and constants values. Correct classification rates were decreased by assigning different weights to rules and the highest classification rate was for *w*_*p*_ = 0.25 for all classes. The results of correct classification rates are displayed in Table 3. After generating rules by system, classification rate was 92.4% by 545 rules which were then reduced to 144 by using expert rule selection; accuracy rate has computed 92.8%.

Multiple logistic regression (MLR) was used for detecting the risk factors effects on CAD (presence or absence). Sensitivity and specificity of MLR were computed, 88.15% and 64.1%, respectively. Also, clinical test PMS in comparison to angiography was found to have sensitivity of 84% and specificity of 92% higher than MLR model.

All patients with CAD by angiography were detected by this proposed fuzzy rule-based classification (Se = 100%) and overall accuracy was 92.8%.

## 4. Discussion and Conclusion

The most important result of this study is the classification of coronary artery disease (CAD) with a high accuracy. Correct classification rate depends on the number of input variables or characteristics, type, and combination of fuzzy rules. We used membership functions for medical variables by reviewing the related literature. These membership functions lead to a high quality of precision in many fields of medicine. We used fuzzy inference method with combination of weighted rules by expert and supervisor pattern classification. This is similar to Chen and Chang study with high accuracy rate (96.88%) in classification [40]. Chen and Fang studied a fuzzy classification method for Iris data with 96.72% accuracy classification rate [41]. They generated fuzzy weighted rules for Iris data with genetic algorithm [42]. The study conducted by Allahverdi et al. for determining the risk factors for CAD had a high corrected classification rate [32]. Vig et al. [31], P. Srivastava and A. Srivastava [33] and Kaya et al. [43] also studied fuzzy set and systems for CAD, cardiac analysis, and congenital heart disease by using fuzzy expert systems.

This study showed that sensitivity and specificity of PMS compared with angiography were acceptable by MLR, but when it was combined with other clinical-epidemiological variables, fuzzy rule-based classification model improved classification rate.

According to the findings of this study, interpretable fuzzy rule-based classification can determine the most important risk factors for CAD and correctly detect the patients who do not need invasive tests such as coronary artery angiography and have a high classification accuracy rate.

## Figures and Tables

**Table 1:** Membership functions for attributes of CAD.

Age	$\mu_{\text{young}}\left(x\right)=\left\{\begin{array}{cc} 1 & x<30\\ \frac{\left(40-x\right)}{10} & 30\leq x<40 \end{array}\right.$
	$\mu_{\text{Middle age}}\left(x\right)=\left\{\begin{array}{cc} \frac{\left(x-30\right)}{10} & 30\leq x<40\\ 1 & 40\leq x<50\\ \frac{\left(60-x\right)}{10} & 50\leq x<60 \end{array}\right.$
	$\mu_{\text{old}}\left(x\right)=\left\{\begin{array}{cc} \frac{\left(x-50\right)}{10} & 50\leq x<60\\ 1 & 60\leq x \end{array}\right.$

Cholesterol	$\mu_{\text{Normal}}\left(x\right)=\left\{\begin{array}{cc} 1 & x<170\\ \frac{\left(200-x\right)}{30} & 170\leq x<200 \end{array}\right.$
	$\mu_{\text{Borderline}}\left(x\right)=\left\{\begin{array}{cc} \frac{\left(x-170\right)}{30} & 170\leq x<200\\ 1 & 200\leq x\leq 225\\ \frac{\left(250-x\right)}{25} & 225\leq x<250 \end{array}\right.$
	$\mu_{\text{High}}\left(x\right)=\left\{\begin{array}{cc} \frac{\left(x-225\right)}{25} & 225\leq x<250\\ 1 & x\geq 250 \end{array}\right.$

Triglyceride	$\mu_{\text{Normal}}\left(x\right)=\left\{\begin{array}{cc} 1 & x<130\\ \frac{\left(200-x\right)}{70} & 130\leq x<200 \end{array}\right.$
	$\mu_{\text{Borderline}}\left(x\right)=\left\{\begin{array}{cc} \frac{\left(x-130\right)}{70} & 130\leq x<200\\ 1 & 200\leq x\leq 300\\ \frac{\left(400-x\right)}{100} & 300\leq x<400 \end{array}\right.$
	$\mu_{\text{High}}\left(x\right)=\left\{\begin{array}{cc} \frac{\left(x-300\right)}{100} & 300\leq x<400\\ 1 & x\geq 400 \end{array}\right.$

LDL	$\mu_{\text{normal}}\left(x\right)=\left\{\begin{array}{cc} 1 & x<110\\ \frac{\left(130-x\right)}{20} & 110\leq x<130 \end{array}\right.$
	$\mu_{\text{middle}}\left(x\right)=\left\{\begin{array}{cc} \frac{\left(x-110\right)}{20} & 110\leq x<130\\ 1 & 130\leq x<145\\ \frac{\left(160-x\right)}{15} & 145\leq x<160 \end{array}\right.$
	$\mu_{\text{High}}\left(x\right)=\left\{\begin{array}{cc} \frac{\left(x-145\right)}{15} & 145\leq x<160\\ 1 & x\geq 160 \end{array}\right.$

Systolic blood pressure	$\mu_{\text{Low}}\left(x\right)=\left\{\begin{array}{cc} 1 & x<105\\ \frac{\left(125-x\right)}{20} & 105\leq x<125 \end{array}\right.$
	$\mu_{\text{Middle}}\left(x\right)=\left\{\begin{array}{cc} \frac{\left(x-105\right)}{20} & 105\leq x<125\\ 1 & 125\leq x\leq 145\\ \frac{\left(170-x\right)}{25} & 145\leq x<170 \end{array}\right.$
	$\mu_{\text{High}}\left(x\right)=\left\{\begin{array}{cc} \frac{\left(x-145\right)}{25} & 145\leq x<170\\ 1 & 170\leq x\leq 180\\ \frac{\left(210-x\right)}{30} & 180\leq x<210 \end{array}\right.$
	$\mu_{\text{Very high}}\left(x\right)=\left\{\begin{array}{cc} \frac{\left(x-180\right)}{30} & 180\leq x<210\\ 1 & x\geq 210 \end{array}\right.$

SSS	$\mu_{\text{Normal}}\left(x\right)=\left\{\begin{array}{cc} 1 & x<2\\ \frac{\left(4-x\right)}{2} & 2\leq x<4 \end{array}\right.$
	$\mu_{\text{Mild}}\left(x\right)=\left\{\begin{array}{cc} \frac{\left(x-2\right)}{2} & 2\leq x<4\\ 1 & 4\leq x<7\\ \frac{\left(9-x\right)}{2} & 7\leq x<9 \end{array}\right.$
	$\mu_{\text{Moderate}}\left(x\right)=\left\{\begin{array}{cc} \frac{\left(x-7\right)}{2} & 7\leq x<9\\ 1 & 9\leq x<13\\ \frac{\left(16-x\right)}{3} & 13\leq x<16 \end{array}\right.$
	$\mu_{\text{Severe}}\left(x\right)=\left\{\begin{array}{cc} \frac{\left(x-13\right)}{3} & 13\leq x<16\\ 1 & x\geq 16 \end{array}\right.$

**Table 2:** Sample fuzzy rules.

Rule number	If										Then	
	Age	CL	TG	LDL	SY	SSS	Sex	DI	SM	GF	Heart disease	*CF*_*j*_
1	M	NR	NR	NR	L	Mo	F	N	Y	N	NR	1.000
2	M	B	B	Mid	L	NR	F	N	N	Y	NR	1.000
3	M	B	B	Mid	Mid	NR	F	N	Y	Y	NR	1.000
4	O	B	B	Mid	Mid	Mi	F	N	N	N	NR	0.941
5	M	B	B	NR	H	Mo	F	Y	N	Y	NR	0.955
6	M	B	B	Mid	Mid	Mo	F	N	N	Y	3.v	1.000
7	O	B	B	Mid	L	S	F	N	N	N	s.v	0.642
8	M	B	B	Mid	Mid	Mi	F	Y	Y	Y	s.v	0.989
9	M	B	NR	Mid	Mid	Mi	M	N	Y	Y	s.v	1.000
10	O	B	B	Mid	H	S	M	Y	N	N	3.v	1.000
11	M	B	NR	NR	L	S	M	N	N	N	2.v	1.000
12	M	B	B	NR	Mid	S	M	Y	Y	Y	2.v	1.000
13	O	H	B	NR	Mid	Mo	M	N	Y	Y	s.v	0.795
14	M	NR	NR	NR	H	S	M	Y	Y	N	3.V	1.000
⋮					⋮							⋮

**Table 3:** Results of correct classification rates with different weighting rules.

*η*	*w*_*p*_ for all classes	Cost(*S*)	Classification rate
*η* = 0.1	(*w*_*p*_ = 0.25 for all classes)	8.5	92.8%

*η* = 0.3	(*w*_*p*_ = 0.25 for all classes)	9.8	91.2%

*η* = 0.1	(*x*_*p*_∈ class 1 *w*_*p*_ = 0.5, *x*_*p*_∈ class 2 *w*_*p*_ = 0.25, *x*_*p*_∈ class 3 *w*_*p*_ = 0.5, *x*_*p*_∈ class 4 *w*_*p*_ = 0.9)	12.4	89.3%

*η* = 0.3	(*x*_*p*_∈ class 1 *w*_*p*_ = 0.5, *x*_*p*_∈ class 2 *w*_*p*_ = 0.25, *x*_*p*_∈ class 3 *w*_*p*_ = 0.5, *x*_*p*_∈ class 4 *w*_*p*_ = 0.9)	19.8	83.6%

*η* = 0.1	(*x*_*p*_∈ class 1 *w*_*p*_ = 0.7, *x*_*p*_∈ class 2 *w*_*p*_ = 0.7, *x*_*p*_∈ class 3 *w*_*p*_ = 0.5 , *x*_*p*_∈ class 4 *w*_*p*_ = 0.25)	15.6	88.9%

*η* = 0.3	(*x*_*p*_∈ class 1 *w*_*p*_ = 0.7, *x*_*p*_∈ class 2 *w*_*p*_ = 0.7, *x*_*p*_∈ class 3 *w*_*p*_ = 0.5 , *x*_*p*_∈ class 4 *w*_*p*_ = 0.25)	18.9	86.4%

*η* = 0.1	(*x*_*p*_∈ class 1 *w*_*p*_ = 0.9, *x*_*p*_∈ class 2 *w*_*p*_ = 0.7, *x*_*p*_∈ class 3 *w*_*p*_ = 0.7 , *x*_*p*_∈ class 4 *w*_*p*_ = 0.5)	26.2	73.1%

*η* = 0.3	(*x*_*p*_∈ class 1 *w*_*p*_ = 0.9, *x*_*p*_∈ class 2 *w*_*p*_ = 0.7, *x*_*p*_∈ class 3 *w*_*p*_ = 0.7 , *x*_*p*_∈ class 4 *w*_*p*_ = 0.5)	30.1	71.8%
